# Lessons and Challenges for Measles Control from Unexpected Large Outbreak, Malawi

**DOI:** 10.3201/eid1902.120301

**Published:** 2013-02

**Authors:** Andrea Minetti, Matthew Kagoli, Agnes Katsulukuta, Helena Huerga, Amber Featherstone, Hazel Chiotcha, Delphine Noel, Cameron Bopp, Laurent Sury, Renzo Fricke, Marta Iscla, Northan Hurtado, Tanya Ducomble, Sarala Nicholas, Storn Kabuluzi, Rebecca F. Grais, Francisco J. Luquero

**Affiliations:** Author affiliations: Epicentre, Paris, France (A. Minetti, H. Huerga, D. Noel, S. Nicholas, R.F Grais, F.J. Luquero);; Ministry of Health, Lilongwe, Malawi (M. Kagoli, A. Katsulukuta, S. Kabuluzi);; Médecins Sans Frontières, Lilongwe (A. Featherstone, H. Chiotcha, C. Bopp);; Médecins Sans Frontières, Paris, France (L. Sury, N. Hurtado);; Médecins Sans Frontières, Brussels, Belgium (R. Fricke, T. Ducomble);; Médecins Sans Frontières, Barcelona, Spain (M. Iscla)

**Keywords:** measles, disease outbreaks, viruses, vaccination, Expanded Program on Immunization, Malawi, control, immunization, vaccines, outbreak

## Abstract

Despite high reported coverage for routine and supplementary immunization, in 2010 in Malawi, a large measles outbreak occurred that comprised 134,000 cases and 304 deaths. Although the highest attack rates were for young children (2.3%, 7.6%, and 4.5% for children <6, 6–8, and 9–11 months, respectively), persons >15 years of age were highly affected (1.0% and 0.4% for persons 15–19 and >19 years, respectively; 28% of all cases). A survey in 8 districts showed routine coverage of 95.0% for children 12–23 months; 57.9% for children 9–11 months; and 60.7% for children covered during the last supplementary immunization activities in 2008. Vaccine effectiveness was 83.9% for 1 dose and 90.5% for 2 doses. A continuous accumulation of susceptible persons during the past decade probably accounts for this outbreak. Countries en route to measles elimination, such as Malawi, should improve outbreak preparedness. Timeliness and the population chosen are crucial elements for reactive campaigns.

## Abstract 2

Supplementary immunization activities are crucial to reduce the number of susceptible children.

During the prevaccine era, 130 million measles cases occurred annually worldwide, and measles was a leading cause of childhood death (*1*). Measles vaccines have dramatically reduced cases and deaths during recent decades. The Measles Initiative developed a joint strategic plan to reduce measles-related deaths by strengthening routine immunization, supplementary immunization activities (SIAs) in the form of mass vaccination campaigns, reinforced surveillance, and adequate case management (*2*,*3*). In 2000, the World Health Organization (WHO) Regional Office for Africa adopted a plan to reduce measles-related deaths by 50% by the end of 2005 (*3*,*4*), and measles-related deaths decreased from 535,300 in 2000 to 139,300 in 2010 (*5*). A recent WHO resolution called for measles elimination in the African Region by 2020 (*6*).

Malawi’s Expanded Program on Immunization (EPI), established in 1979, recommends 1 dose of measles-containing vaccine (MCV) for infants 9–11 months of age (*7*). After implementation of EPI, cases declined from >162,000 in 1980 to an annual average of 8,000 cases throughout the 1990s. Additional initiatives toward measles control (*8*) comprised a catch-up campaign in 1998 directed toward children 9 months–14 years of age and follow-up campaigns in 2002, 2005, and 2008 for children 9–59 months (*9*); administrative reported vaccine coverage was close to 100% (*10*). Before 2010, the last large epidemic in Malawi occurred in 1996 and 1997, when ≈10,000 cases were reported nationwide each year.

Despite Malawi’s measles control successes during the past 2 decades, a large outbreak occurred in 2010, with as many cases as in the 1980s. Médecins Sans Frontières (MSF) with the Ministry of Health (MoH) reinforced surveillance, provided case management, and vaccinated 3,343,112 children through outbreak resonse immunization (MoH/MSF) during epidemiologic weeks 18–26 (May–June) (Figure 1, panel A). The MoH implemented additional reactive vaccination campaigns for 1) children 9–59 months of age in some districts in epidemiologic weeks 10–14 (March–April) and 2) children 9 months–15 years of age nationwide in epidemiologic weeks 33–34 (August).

**Figure 1:**
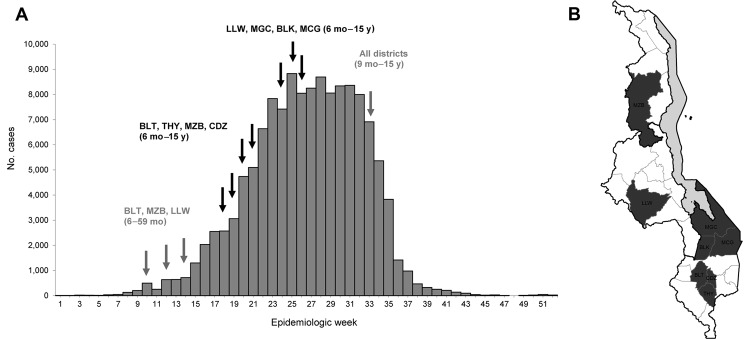
Weekly distribution of measles cases and time when the reactive vaccination campaigns were implemented (A) and districts where outbreak-response immunizations were conducted by the MoH and MSF (B), Malawi, 2010. MoH, Ministry of Health; MSF, Médecins Sans Frontières; BLT, Blantyre; MZB, Mzimba, LLW, Lilongwe; THY, Thyolo; CDZ, Chiradzulu; MGC, Mangochi; BLK, Balaka; MCG, Machinga. Black arrows indicate campaigns implemented by MoH/MSF; gray arrows indicate campaigns implemented by MoH only.

To describe this epidemic and outcomes from outbreak response vaccination, we analyzed national surveillance data. We also addressed factors that might explain the outbreak, including results from coverage and vaccine effectiveness surveys.

## Field Studies

### Measles Surveillance

National surveillance in Malawi is based on the Integrated Disease Surveillance and Response strategy. Each month, health officers from public health facilities and selected private centers report cases and deaths to the district level. Data are electronically compiled and transmitted monthly to the national level. A standardized individual collection form is used to collect information about age, sex, health facility, date of consultation, date of onset, whether specimen was sent to the laboratory, treatment, and outcome (alive or dead).

Health care providers used WHO case definitions to diagnose measles cases and to report case-patients in the surveillance system. Suspected measles was defined as generalized maculopapular rash and fever (>38°C) and at least 1 of the following: cough, runny nose, or conjunctivitis; or as suspected measles reported by a health professional. A measles-related death was defined as the death of a person with measles within 30 days after rash onset, unless the death was unrelated to the disease. Samples from suspected case-patients were sent for laboratory confirmation to detect measles IgM. Three confirmed cases at the district level constituted an outbreak. Once an outbreak was confirmed, additional cases were confirmed by epidemiologic link if they met the clinical case definition (*1*).

During the 2010 outbreak, the surveillance system was strengthened by reinforcing health officer training (case definition and data collection), retrospective review of health registers, weekly communication to the district level for data sharing, monitoring of data completeness, and electronic compilation and cleaning of the line list. Data collection strengthening was interrupted in epidemiologic week 35 when MSF involvement in measles case management ended.

Data were entered in Excel (Microsoft, Redmond, WA, USA). Attack rates (ARs) were defined as the number of measles cases divided by the population at risk and case-fatality rates (CFRs) as the number of measles-related deaths divided by the number of measles cases. Population projections for 2010 were calculated by applying a growth rate of 3.28% to the 2008 Malawi Population and Housing Census figures.

### Vaccine Coverage and Effectiveness

We conducted age-stratified surveys (epidemiologic weeks 27–31 [July–August 2010]) to assess coverage in the districts where MoH/MSF conducted vaccination campaigns (Figure 1, panel B). Children 6 months–15 years of age were eligible for inclusion in the surveys. For each district, we required a sample of 983 on the basis of the following assumptions: 80% vaccine coverage, an α error of 0.05, an absolute precision of 0.05, and a design effect of 4.

We used cluster sampling (40 villages of 25 children in each district); clusters were allocated proportionately to the population of each village or health center catchment area where village populations were not available. In the urban areas of Blantyre and Lilongwe, spatial-based sampling was used to select randomly the first household of clusters (*11*). In rural areas, the households in the villages were numbered, and the first household was randomly selected. Subsequent households were selected by proximity. Households were defined as persons living and eating together under the same roof. If a head of household or adult was absent, the survey team returned later in the day; if a head of household was absent after 2 attempts, the household was skipped and replaced.

A standardized questionnaire was used to collect demographic data, vaccination status, and vaccination history (place, date of vaccination, and injection site on the body) for routine vaccination, last SIAs (October 2008) and MoH/MSF campaigns (May–July 2010), reasons for nonvaccination for routine vaccination and MoH/MSF outbreak response immunization, and previous measles episodes. Proof of vaccination status was verified in the health passport; otherwise we relied on verbal recall. We also asked for respondents’ age and degree of literacy.

To estimate vaccine effectiveness, we identified all children born after October 2003 (oldest age group toward which the 2008 follow-up SIAs were directed) with known vaccination status and no previous measles episode before 2010 among those recruited for the vaccine coverage survey. The main exposure of interest was vaccination status. Children were classified as not vaccinated, vaccinated with the routine dose only (routine vaccination ascertained by health passport), or vaccinated with 2 doses (routine vaccination ascertained by health passport plus the 2008 SIAs). Case-patients were defined as any child 9 months–15 years of age whose illness met the WHO measles case definition from January 2010 to the date of MoH/MSF outbreak response immunization in the different districts. Additional variables considered as possible confounders were age and literacy of the main caregiver and district of residence.

Vaccination coverage estimates were obtained by taking into account the survey design; sampling weights and design effect (deff) were applied to obtain the estimates (*12*). Associations between vaccination status and outcome were assessed through binomial regression (log link). The exponential of the coefficient for the vaccination variable was computed to estimate the adjusted risk ratio, and p values <0.05 were regarded as significant. The adjusted level of vaccine protection was computed as follows: (1 − adjusted risk ratio) × 100. Confidence intervals were calculated by taking into account the deff.

Data were entered by using EpiData 3.1 (EpiData Association, Odense, Denmark). Data analysis was performed by using Stata 10.0 (Stata Corp., College Station, TX, USA).

### Ethical Considerations

The study was implemented in collaboration with the MoH after obtaining authorization. Privacy, confidentiality, and rights of patients were ensured during and after the study. Verbal informed consent was obtained from each head of household visited after detailed explanation about the existence of the outbreak, study objective, and planned use of the data collected. The survey data were entered and analyzed anonymously.

## Main Findings

### Description of the Outbreak

In January 2010, sporadic suspected measles cases were reported in Blantyre and Zomba districts (Southern Region) and in Mzimba and Nkhata Bay districts (Northern Region). The first samples were confirmed measles IgM positive on week 3. For the first few weeks, most reported cases were from the urban area of Blantyre, but by the end of March, all 3 regions were affected; the bulk of cases came from a few districts: Blantyre, Chiradzulu, Mangochi, Machinga, Zomba, and Thyolo (Southern Region); Mzimba (Northern Region); and Lilongwe (Central Region). By mid-July, all districts reported measles cases.

During weeks 1–52, a total of 134,039 measles cases and 304 deaths were reported. At the national level, the epidemic plateaued on week 23, and reported cases started to decrease in week 32 (Figure 1). The overall cumulative AR was 0.96%. The CFR was 0.23%.

**Figure 2:**
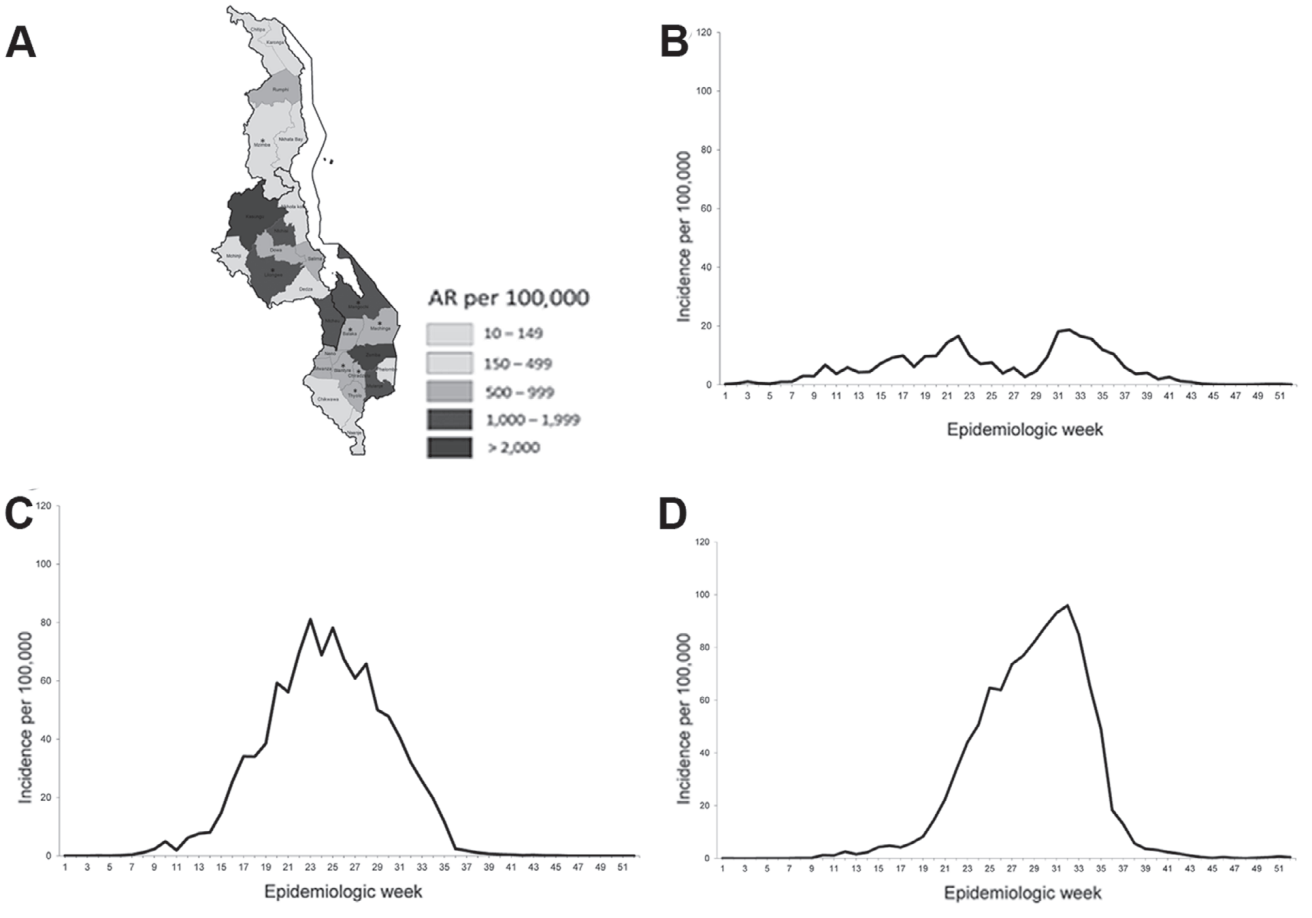
Measles cumulative attack rates (ARs) by district and weekly incidence, in Malawi, 2010. A) Malawi. The white area is Malawi Lake. Asterisks indicate districts in which children were vaccinated. B) Northern Region. C) Southern Region. D) Central Region.

The most affected areas were the Central Region and the Southern Region, where cumulative ARs were 1.09% and 1.03%, respectively; in the Northern Region, the AR was 0.28%. In the Southern Region, cases peaked in week 23, and in the Central Region, in week 32 (Figure 2).

**Figure 3:**
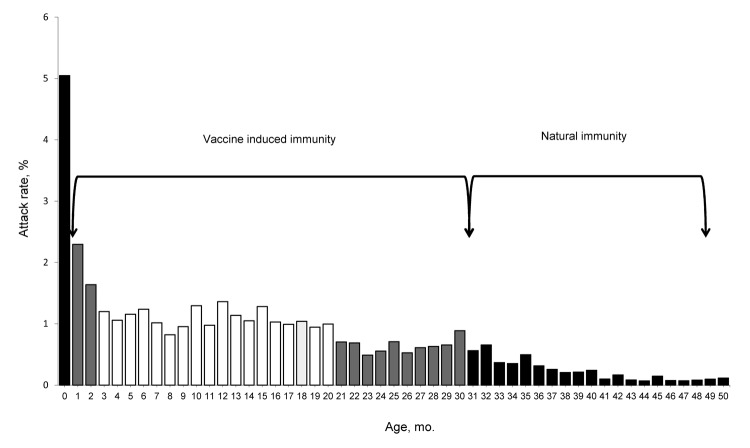
Age distribution of children with measles reported in Malawi, 2010. Vaccine-induced immunity of children in age groups vaccinated mostly through a 2-dose vaccination strategy, whether they have been offered 1 dose of vaccine (2 years, 21–30 years of age) or 2 doses of vaccine (3–20 years of age) and natural immunity of children in age groups mostly immunized through natural infection (>31 years of age). Black bars, no vaccine offered; gray bars, 1 vaccine dose offered; white bars, 2 vaccine doses offered.

Measles cases were equally distributed between male and female patients (M:F ratio 1.03:1) (Table 1). Median age of case-patients was 7 years (interquartile range 1–16). A total of 54,138 case-patients were <5 years of age, constituting 42% of all cases; 30% of case-patients were 5–14 years old, and 28% were adults (>15 years). The most affected age groups were infants <1 year of age; AR was highest among children 6–8 months of age, followed by those 9–11 months (Figure 3).

**Table 1:** Characteristics of persons reported to have measles, Malawi, 2010

Characteristic	No. (%) cases reported	Attack rate*	No. (%) deaths reported	Case-fatality rate†
Total Malawi	134,039 (100)	0.96	304 (100)	0.23
Northern Region	5,054 (4)	0.28	24 (8)	0.47
Central Region	64,688 (48)	1.09	152 (50)	0.23
Southern Region	64,297 (48)	1.03	128 (42)	0.20
Sex	133,834 (100)		304 (100)	
M	67,949 (51)	0.97	126 (41)	0.19
F	65,885 (49)	0.94	178 (59)	0.27
Age group	131,725 (100)		292 (100)	
0–5 mo	7,243 (6)	2.26	10 (3)	0.14
6–8 mo	10,615 (8)	7.61	27 (9)	0.25
9–11 mo	7,543 (6)	4.5	21 (7)	0.28
12–59 mo	28,737 (22)	1.38	81 (28)	0.28
5–14 y	39,979 (30)	1.02	69 (24)	0.17
15–19 y	13,641 (10)	1.0	14 (5)	0.10
>20 y	23,967 (18)	0.4	70 (24)	0.29

*Per 100 persons.  †Per 100 measles cases.

### Vaccine Coverage and Effectiveness

A total of 9,381 households were visited, and 21,993 children participated in the surveys. The median age of children was 6.8 years (interquartile range 3–10 years).

**Figure 4:**
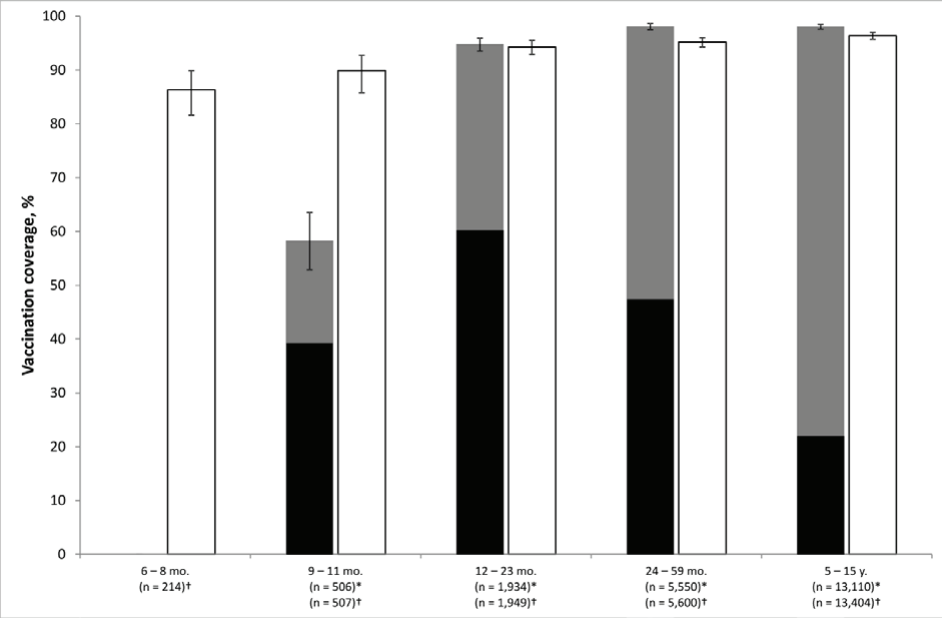
Measles vaccine coverage for the Expanded Program on Immunization and outbreak-response immunization conducted by the Ministry of Health and Médecins Sans Frontières in 8 districts (Blantyre, Mzimba, Lilongwe, Thyolo, Chiradzulu, Mangochi, Balaka, and Machinga), Malawi, 2010. *EPI; †ORI; black bars, vaccination ascertained by health passport; gray bars, EPI vaccination ascertianed by oral reporting; white bars, ORI vaccination ascertained by oral reporting.

In the 8 districts surveyed, 95.0% (95% CI 93.7%–96.0%, deff 1.5) of children 12–23 months of age were vaccinated through EPI (Figure 4). A similar percentage (96.9% [95% CI 96.4%–97.3%, deff 4.0]­), was observed overall. Reason for nonvaccination was obtained for 661 (88.6%) children not vaccinated through EPI. Caregivers decided not to vaccinate the child in 61.8% of instances; the rest were unable to vaccinate the child despite their willingness because of reasons such as distance and cost. The main reason for nonvaccination was the caregiver’s belief that the child was too young (36.0%); few (4.4%) declared religious objection to vaccination.

As reported by the caregiver, 60.8% (95% CI 58.3%–63.1%, deff 5.7) of children 9 months–5 years of age at the time of the SIA in 2008 were vaccinated against measles during this SIA; 0.7% of these children received the first dose, 60.1% received the second dose, and 39.2% were not vaccinated. Children not included in the age group toward which immunization was directed were vaccinated during the SIA (11% of children 0–9 months and 19% of children 5–10 years). Among the 159 (1.7%) children 9 months–5 years of age not vaccinated through EPI by October 2008, 54 (34.8%) were vaccinated in the SIA.

Coverage of the MoH/MSF outbreak response immunization was 95.5% (95% CI 94.9%–96.1%, deff 4.7) and lowest in children 6 months–1 year of age (Figure 4). Results were similar across the 8 districts surveyed (Table 2).

**Table 2:** Measles vaccination coverage estimates in 8 districts, Malawi, 2010*

Vaccination coverage, age group	Estimates, % (95% CI)						
	Blantyre	Chiradzulu	Lilongwe	Mzimba	Thyolo	Mangochi	Bal-Mach
Routine EPI vaccination, 9 mo–15 y	98 (96–98)	99 (98–99)	95 (92–97)	95 (93–97)	97 (96–98)	96 (95–97)	98 (97–99)
MoH/MSF reactive campaign, 6 mo–15 y	93 (91–94)	98 (97–98)	96 (94–97)	92 (90–94)	97 (95–97)	96 (95–98)	96 (94–97)
SIA October 2008, 9 mo–5 y	47 (40–54)	57 (52–63)	60 (55–65)	47 (41–53)	69 (64–74)	63 (57–68)	6 (63–74)


*Bal-Mach, Balaka-Machinga; EPI, Expanded Program on Immunization; MoH/MSF, Ministry of Health/ Médecins Sans Frontières; SIA, supplementary immunization activity.

In total, 175 (3.4%) children among the 5,082 in the effectiveness survey reported a measles episode in 2010 before the MoH/MSF outbreak response immunization was implemented. Most (90.8%) children consulted in a health facility, and 21.8% were hospitalized. The vaccine effectiveness adjusted by child’s age, sex, and location and the main caregiver’s literacy was 83.9% (95% CI 70.8%–90.8%) for 1 EPI dose and 90.5% (95% CI 79.7%–95.5%) for 2 doses (EPI and SIAs) (Table 3). Vaccine effectiveness was highest among children 1–2 years, but effectiveness did not significantly differ by age group (data not shown).

**Table 3:** Results from the vaccine effectiveness study in 8 districts, Malawi, 2010*

Vaccination status	Total	No. cases	Attack rate, %†	Vaccine effectiveness	
				Crude, % (95% CI)	Adjusted, % (95% CI)‡
Unvaccinated	793	91	13.3	Referent	Referent
Vaccinated with MCV					
1 dose§	2512	62	2.4	82.0 (72.9–88.1)	83.9 (70.8–90.8)
2 doses¶	1777	22	1.1	91.6 (84.7–95.4)	90.5 (79.7–95.5)

*Districts are Blantyre, Mzimba, Lilongwe, Thyolo, Chiradzulu, Mangochi, Balaka, and Machinga. MCV, measles-containing vaccine. †Weighted and adjusted by the study design. ‡Adjusted by age, sex, and literacy of the caregiver. §Routine immunization. ¶Routine immunization and 2008 supplementary immunization activities.

## Lessons Learned

In November 2009, WHO and the Global Alliance on Vaccination and Immunization rewarded the Malawi MoH for its outstanding performance in improving child health and immunization. The measles control program in Malawi is, to a large extent, a success story. However, despite high reported coverage for both EPI and SIAs, implemented in recent years in a timely manner, in 2010 Malawi faced its largest measles outbreak in >2 decades, with a number of reported cases comparable to the prevaccine era.

Malawi reported fewer deaths from measles than did other African countries where measles outbreaks recently occurred (*13*–*15*). Although measles-related deaths are known to be underreported, perhaps the best explanation for low numbers of reported deaths is the high measles vaccine coverage combined with the lower CFR among vaccinated case-patients (*16*,*17*). In addition, a high proportion of cases occurred in older children and young adults, who are at lower risk for death (*18*–*20*). The wide age distribution documented in this outbreak corresponds to a setting with good coverage and adequate vaccine effectiveness but insufficient to achieve elimination (*18*).

The EPI measles coverage (first dose of MCV) was high according to administrative estimates and from the results of vaccine coverage surveys in selected districts. Nonetheless, children 9–11 months of age were one of the most affected age groups during the 2010 outbreak. The routine vaccine coverage was low for this group, suggesting that children are vaccinated toward the end of or after the recommended period for EPI. The main reason for nonvaccination was the belief that the child was too young to be vaccinated. This issue highlights the need to reinforce routine vaccination, even in high-performing programs such as that in Malawi, and to include clear advice on the age for vaccination (*19*). In Malawi, the first measles dose is recommended at 9–11 months; however, older children also should be vaccinated through the routine program if they have not been vaccinated at the recommended age.

ARs during the 2010 outbreak were highest for age groups not yet vaccinated or for children who had received only 1 dose of MCV (i.e., children born after the last SIAs in October 2008). However, ARs also were relatively high for age groups eligible for SIAs. The reported administrative coverage of the last SIA in 2008 was 98% (*10*), but survey results indicate that only 61% of children eligible for the SIA in 2008 were vaccinated. The discrepancy between survey figures and the administrative coverage might be explained by denominator problems (administrative coverage), by possible recall bias (survey coverage), and by the fact that children out of the age groups toward which routine vaccination is directed are vaccinated during the SIA as documented in our survey. Most children vaccinated during the SIA already had been vaccinated through EPI; the SIA captured only one third of those not yet vaccinated.

We estimated the vaccine effectiveness for 1 dose to be 84%, meaning that each year 16% of children vaccinated through the routine program in Malawi are not protected. This percentage is expected for a vaccination that recommends giving a first dose to children at 9 months of age (the seroconversion rate at this age is 85%–90%) (*20*) but is insufficient to prevent measles outbreaks, as was shown in Malawi. One option for increasing vaccine effectiveness is to provide the first measles dose at 12 months, when seroconversion is more likely (*21*). WHO recommends this strategy in the absence of large outbreaks and documented good coverage. Nonetheless, when this schedule is followed, the children remain susceptible to measles infection for an additional 3 months, and in countries with a high birth rate (such as Malawi), this schedule may result in the buildup of a large cohort of susceptible children. Because the youngest children are at higher risk for measles-related death (*17*), careful consideration must be given to any increase in the age at first measles vaccination, especially if the risk for an outbreak remains high (*22*).

Reaching all children with 2 doses of MCV should be the standard for all national immunization programs—especially in countries with an elimination goal—to increase the seroconversion rates (*20*). However, in our study the estimated effectiveness of the second dose was lower than expected; for children receiving the second measles vaccine dose through the 2008 SIA, vaccine effectiveness was 91%. Factors that can reduce vaccine protection include failure in the cold chain, interaction with maternal antibodies, waning immunity, and the HIV pandemic (*23*). The vaccine is less effective in HIV-infected children because of quick loss of protective antibodies after immunization (*24*). In Malawi, ≈1%–2% of children <15 years of age are HIV infected (*25*). WHO recommends vaccination at 6 months in addition to the normal schedule in areas with high HIV prevalence and measles transmission (*20*), and incorporating this guidance into the routine program remains a priority.

Despite several reactive vaccination campaigns, the epidemic spread throughout Malawi. WHO guidelines on measles outbreak response suggest that reactive vaccination should be implemented as quickly as possible; at-risk groups and affected areas also should be considered as areas that are not yet affected but have high epidemic risk (*1*). The MoH campaign conducted early during the outbreak (March–April) was not wide enough in terms of focus population and geographic extension to contain the epidemic. The MoH/MSF nonselective campaigns were implemented in several districts for children 6 months–15 years of age, but more than one third of cases were reported among older persons. Despite overall high vaccine coverage obtained without major differences across districts, most of these campaigns were implemented late in the epidemic (during or after the peak) (Technical Appendix Figure). In addition, campaigns were conducted in some of the most affected districts but not in neighboring districts with high transmission. The national mass vaccination campaign conducted by the MoH in August (reported administrative vaccine coverage of 107%) also was implemented late in the course of the epidemic and also focused on persons <15 years of age. The timeliness of the intervention is probably the major factor determining the effect of the outbreak response; the delay in Malawi probably decreased the effect of the immunization campaigns. Our experience in 2010 shows that better preparedness plans for outbreak response are needed to improve the timeliness of such interventions. Prior evidence favored conducting timely reactive campaigns directed toward children 6 months–15 years to maximize cases averted (*26*). However, in areas such as Malawi, where older age groups are highly affected, efforts directed toward persons >15 years of age would have had a greater effect on the transmission dynamics.

Our field studies have some key limitations. First, despite the effort to detect and report all suspected measles cases and related deaths to the national surveillance system, only case-patients seeking treatment at health facilities were recorded. Thus, the actual number of measles cases and measles-related deaths remain unknown. In addition, completeness of case reporting and sensitivity of the surveillance system were not assessed. However, health services are accessible and functional in Malawi, and free treatment for measles was provided during the epidemic; moreover the survey results showed that most of the children were vaccinated at a health facility. Despite the effort to standardize procedures for data collection in all districts, active surveillance was reinforced more in districts where case management was implemented by MSF. Thus, differences in number of cases reported might partially reflect the difference in performance of the surveillance system.

Second, we conducted surveys only in districts where reactive vaccination campaigns were implemented, which makes extrapolation to the country difficult. In addition, because not all children had health passports, vaccination coverage was assessed by parental recall, leading to possible overestimation or underestimation of coverage. To minimize misclassification of verbally reported vaccination status, we asked parents to name the vaccination place and the part of the body where the vaccine was delivered (e.g., shoulder, leg, other) to determine whether the caregiver correctly remembered a vaccine consistent with measles vaccine delivery. Previous studies in areas of high measles incidence have shown parental recall to be reliable (*27*). We suspect underreporting for the 2008 SIA given that all the information for this estimate was collected from verbal reporting because documentation was not available and the recall period was long.

Third, misclassification of SIA vaccination status might have decreased the vaccine effectiveness estimate for the second dose (*28*) and should be considered as an additional reason to explain the lower-than-expected vaccine effectiveness for 2 doses. Regarding the retrospective ascertainment of measles cases, we used the local term for measles to increase the sensitivity and specificity of the case definition (*29*). However, if misclassification is present, it probably does not differ for vaccinated and nonvaccinated children. Finally, we did not collect information about measles-related deaths, which might slightly downward bias our estimate of vaccine efficacy.

Measles and measles-related deaths have decreased dramatically during recent decades in Malawi thanks to a comprehensive measles control strategy. However, our results highlight the difficulties in avoiding large outbreaks, even with successful routine programs. Control programs need to be adapted to the epidemiologic context, including age range for routine vaccination. SIAs are crucial for reducing the number of susceptible children. This SIA strategy has been successfully implemented in the Americas, which achieved measles elimination in 2003, but reality shows the complexity of obtaining similar outcomes in other locations. SIAs could focus on a wider age range if older nonvaccinated persons are expected. We also highlight the need for the timeliness and choice of the population outbreak response immunizations. Age groups toward which vaccination efforts are directed should be determined according to local measles epidemiology. To provide timely and adequate responses in similar contexts, better preparedness plans for possible outbreaks based on proper risk assessments, including good estimates of vaccine coverage, are urgently needed.

Technical Appendix Weekly incidence and timeliness of the outbreaks response immunizations conducted by the Ministry of Health and Médecins Sans Frontières, Malawi, 2010.
