# Supplementary material for: Lessons and Challenges for Measles Control from Unexpected Large Outbreak, Malawi
Source: Emerg Infect Dis. 2013 Feb;19(2):202–9. doi: 10.3201/eid1902.120301 (PMC3559033; doi:10.3201/eid1902.120301)
Supplement: Technical Appendix — Weekly incidence and timeliness of the outbreaks response immunizations conducted by the Ministry of Health and Médecins Sans Frontières, Malawi, 2010. [file 12-0301-Techapp-s1.pdf]

Article DOI: http://dx.doi.org/10.3201/eid1902.120301

Lessons and Challenges for Measles Control from Unexpected Large Outbreak, Malawi

Technical Appendix

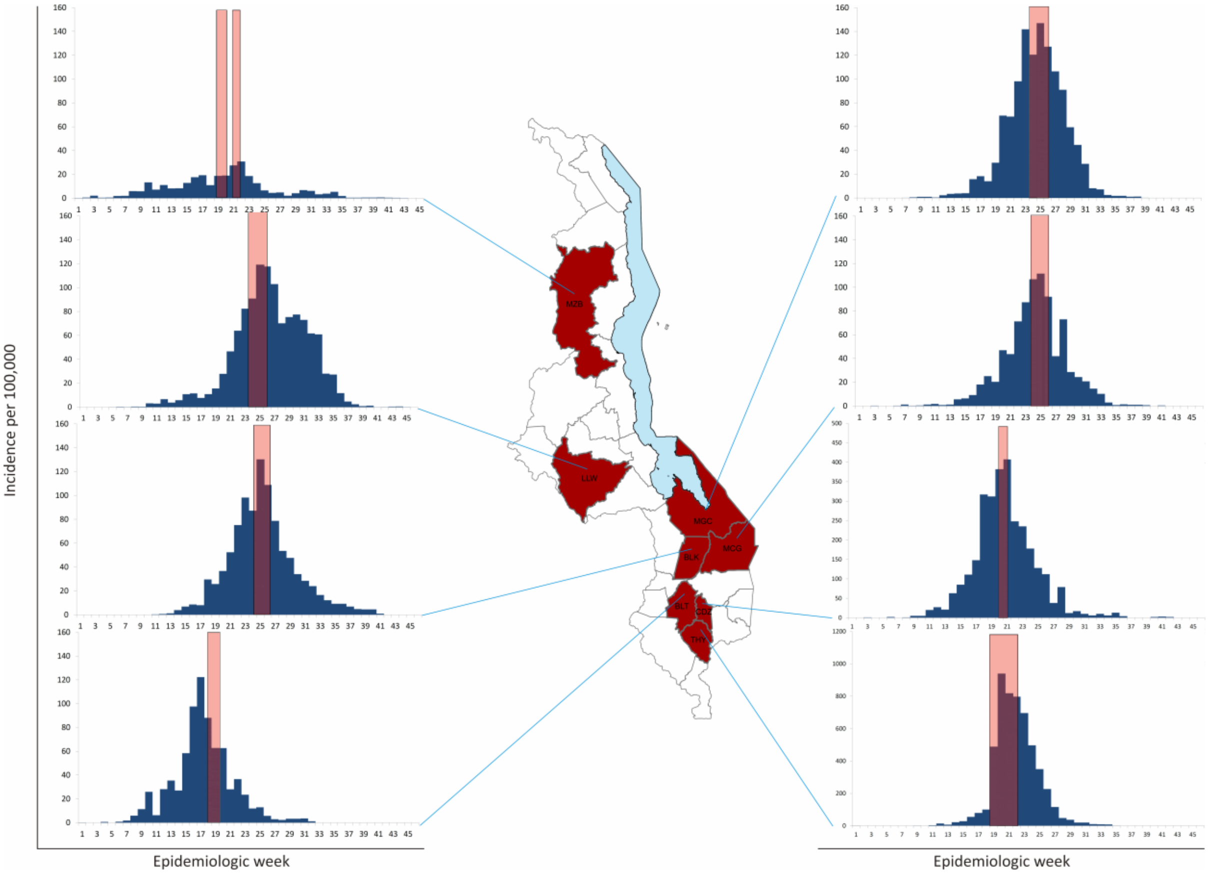

Technical Appendix Figure. Weekly incidence and timeliness of the outbreaks response immunizations conducted by the Ministry of Health and Médecins Sans Frontières, Malawi, 2010. BLT, Blantyre; MZB, Mzimba; LLW, Lilongwe; THY, Thyolo; CDZ, Chiradzulu; MGC, Mangochi; BLK, Balaka; MCG, Machinga.
